# Differential effect sizes of growth hormone replacement on quality of life, well-being and health status in growth hormone deficient patients: a meta-analysis

**DOI:** 10.1186/1477-7525-3-63

**Published:** 2005-10-19

**Authors:** Jan Berend Deijen, Lucia I Arwert, Joost Witlox, Madeleine L Drent

**Affiliations:** 1 Department of Clinical Neuropsychology, Free University, van der Boechorststraat 1, 1081 BT Amsterdam, the Netherlands; 2 Department of Endocrinology, VU University Medical Center, PO Box 7057, 1007 MB, Amsterdam, the Netherlands

## Abstract

**Background:**

Patients with growth hormone deficiency (GHD) frequently report to suffer from an impaired Quality of Life (QoL) and growth hormone (GH) substitution is found to improve this. However, the same test may be used for measuring QoL, well-being or health status in different studies. QoL has been defined as the subjective appraisal of one's current life based primarily on psychological function. The most important in the appraisal of well-being is mental function and concerning health status patients evaluate physical function as most important. To differentiate the effects of GH replacement on psychological variables in patients with GHD we carried out a number of meta-analyses, classifying questionnaires into instruments measuring QoL, psychological well-being and health status.

**Methods:**

We searched the electronic databases PUBMED and PiCarta from 1985 to 2004. Studies were included that evaluated the effect of GH on patient-reported outcomes in adults with GHD (aged 18 years and above). According to generally accepted definitions we classified the questionnaires as instruments measuring QoL, well-being and health status. By means of meta-analyses the average effect size (*d*) for QoL, well-being and health status was calculated.

**Results and Discussion:**

Based on open studies GH replacement is found to improve QoL with a small effect size (*d *= 0.18), well-being with a medium effect size (*d *= 0.47) and health status with a small effect size (*d *= 0.26). As the effect size of well-being is most pronounced the generally reported effects of GH replacement on QoL may be overestimated and actually reflect the effect on well-being.

**Conclusion:**

To get more insight in the specific psychological effects of GH treatment it is recommended that instruments selected for these studies should be more consistently classified as instruments measuring QoL, well-being or health status.

## Background

The concept of Quality of life (QoL) is frequently used in reports of studies in patients with growth hormone deficiency (GHD). It is already 4 decades ago that the first report was published on QoL in relation to growth hormone deficiency. In this particular study the effect of GH substitution on QoL in a GHD patient is described [1]. From then on, it has been frequently reported that patients with GHD suffer from an impaired Quality of Life (QoL), and that growth hormone (GH) substitution improves their condition [2-6]. With regard to GHD, a subnormal QoL in adults with GHD is inferred from the observations that these patients feel less energetic, are emotionally more labile, and experience disturbances in sex life and feelings of social isolation at a significantly higher frequency than controls [2,3,6,7]. With respect to GH substitution in GHD adults, the effects of 10 years of GH replacement on psychological well-being have been evaluated. Overall scores for the NHP, energy levels and emotional reaction improved in the GH-treated group compared to an untreated group [8]. In addition, one year of discontinuation of GH treatment in a study in GHD patients led to a decrease in QoL (psychological complaints and depression). This effect was counteracted after restart of GH therapy resulting in reduced anxiety and depression and improved QoL [9]. In another study withdrawal of GH treatment from adults with GHD had detrimental psychological effects (decreased energy, increased tiredness, pain, irritability and depression) [10]. The studies above exemplify that the psychological effects of GH replacement are being measured with a variety of instruments. Moreover, the same tests may be used as an instrument measuring QoL in one and well-being or health status in another study.

The concept of QoL with particular relevance to patients with GH deficiency has been defined as "the social and psychological well-being assessed from the patient's perspective". Elements which contribute to a person's QoL are their levels of emotional, cognitive and social functioning [11]. Indeed, it is generally acknowledged that the QoL of a patient is not only defined by quantitative factors of a disease, for example the severity of GH deficiency, but also by psychosocial factors. Functional impairments, not being able to perform personal goals, unemployment and relational problems should also be taken into account when measuring the influence of a disease for a patient [12].

A decade ago editorial attention in the Lancet was paid to the conceptual and methodological difficulties pertaining to the concept of QoL [13]. This editorial included the study of Gill and Feinstein [14] who sampled 75 articles with "quality of life' in their titles. Only 15% of the sample included definitions of QoL and in only 13% of the cases patient-rated QoL measures – as opposed to 'objective' questionnaires – were used. From the perspective of patients, QoL and health status have been found to be distinct constructs. QoL has been defined as 'the subjective appraisal of one's current life based primarily on psychological function and to a lesser degree on physical functioning'. Indeed, when rating QoL, patients give greater emphasis to mental health than to physical functioning. This pattern is reversed for appraisal of health status, for which physical function is more important than mental health [15]. In a second commentary in the Lancet the importance of differentiating health status from QoL is also addressed. It is stated that using a health status questionnaire, which is measuring how people feel about their health, to assess QoL may lead to misleading conclusions. In case of health status patients report how they feel mainly about their physical health, whereas in case of reporting well-being patients exhibit feelings of depression, anxiety and energy [16]. Thus, patient reported outcomes of physical status may be specifically measured by a health status questionnaire such as the Short-form Health Survey (SF-36) [17], whereas mental status may be measured by a well-being questionnaire such as the Psychological General Well-being Index (PGWB) [18]. It may be clear that such generic measures of health status and well-being are not measures of QoL, although often categorized as such. Therefore, with respect to the measurement of QoL in patients with GHD, a few disease-specific QoL questionnaires have been developed, the QoL-AGHDA being one of these. This is a condition-specific QoL measure. All items of this instrument are expressed as unsatisfied needs [19]. More recently, the psychometric properties of a new individualized questionnaire, the A-RHDQoL, measuring perceived impact of age-related hormonal decline on QoL in older men, have been reported. The questionnaire is individualized because respondents only rate those domains that are relevant to them. Both the impact of age-related hormonal decline on life domains and the importance of each domain to the individual are taken into account [10].

A complete picture of the effects of GH replacement on psychological status in GHD patients can be inferred from changes determined with a QoL scale, in conjunction with an established measure of health status and another of well-being. However, up until now these scales have not been used consequently to measure specifically one of these concepts. As a consequence, in spite of a number of studies on the effects of GH treatment on psychological status, the relative contribution of GH treatment on changes in QoL, well-being and health status is not known yet.

In order to differentiate the effects of GH replacement on psychological variables in patients with GHD we carried out a number of meta-analyses, distinguishing between QoL, psychological well-being and health status.

## Methods

### Search strategy

We searched the electronic databases PUBMED and PiCarta from 1985 to 2004. PiCarta is an integrated multimaterial database with request-facilities and offering access to online resources and electronic documents.

Studies were included that evaluated the effect of GH on patient-reported outcomes in adults with GHD (aged 18 years and above). The following search terms were used: growth hormone, mood, health status, well-being and quality of life.

### Study selection

Two investigators independently examined manuscripts for inclusion. Eligible studies were reports providing quantitative data about the effect of GH therapy on patient-reported outcomes in GH deficient adults. Studies had to be placebo-controlled or designed as a cross-over/parallel or open clinical trial. Questionnaires had to be used to measure patient-reported outcomes. Case reports, review articles and studies in which the psychometric quality of the used questionnaire was unknown were excluded. Furthermore, studies on GH therapy for other diseases (for instance Turner syndrome, Prader Willi Syndrome, fibro-myalgia, etc.) were not included in this meta-analysis.

### Statistical analysis

We carried out a series of meta-analyses using a random effects model. The meta-analyses were performed by means of the statistical package C*omprehensive Meta-analysis (Biostat, Inc, USA) *[20]. This program is used to determine *d*-values (effect sizes). The most commonly used measures of effect size are the standardized mean difference (*d*) and the correlation coefficient (*r*). The effect size is a simple quantitative measure that provides one useful index of the importance of an effect. The effect size index *d *standardizes the raw effect size as expressed in the measurement unit of the dependent variable by dividing it by the common SD of the measures in their respective populations [21]. We calculated the difference prior to and after GH therapy, divided by the pooled standard deviation of the two measurements. Effect sizes (*d*'s) were calculated, averaged for each study and pooled. Effect size *d *= 0.2–0.5 is conceived as a small effect, *d *= 0.5–0.8 as a medium effect and *d *≥ 0.8 as a large effect. A medium effect size is conceived as one large enough to be visible to the naked eye [21].

### Questionnaires

The most frequently encountered questionnaires measuring QoL, well-being or health status encountered in the studies included in the meta-analysis are described below.

The *Nottingham Health Profile *(NHP) is a frequently used health status questionnaire in GH deficient patients that measures physical, emotional and social distress. It consists of the subscales emotional reactions, energy, pain, physical mobility, sleep and social isolation [22]. The *Psychological General Well Being Schedule *(PGWB) measures self-perceived affective and emotional states [18]. Subscales include anxiety, depressed mood, positive well-being, self-control, general health and vitality. The *Hopkins Symptom Checklist *(HSCL) is a questionnaire for the assessment of psychological and somatic complaints [23]. The *Profile of Mood States *(POMS) is a 32-item questionnaire with subscales depression, anger, fatigue, vigor and tension [24] and the *State-Trait Anxiety Inventory *(STAI) is a questionnaire to assess state and trait anxiety [25]. The *Quality of Life Assessment of Growth Hormone Deficiency in Adults (QoL-AGHDA) *[19] is especially designed to assess relevant aspects of GHD.

### Distinguishing between QoL, well-being and health status

As is pointed out above, QoL is conceived as the total of psychosocial determinants and physical functioning assessed from the patient's perspective. We made the QoL concept operational by "the subjective judgment of the quality of daily functioning related to psychological or physical capabilities" and classified the questionnaires accordingly as QoL. In addition, as well-being is perceived as feelings of depression, anxiety and energy, and health status as feelings about physical health we defined well-being as perceived mental health and health status as perceived physical health. According to the above criteria we classified the questionnaires into the three categories. If instruments have multiple domains measuring QoL, well-being or health status, we classified these domains separately. The result of our classification is summarized in Table 1.

**Table 1:** Classification of questionnaires into instruments measuring quality of life, psychological well-being or health status

Quality of Life	• Qol Assessment of Growth Hormone Deficiency in Adults (QoL-AGHDA)
	• Satisfaction with physical activity (VAS-score)
	• Sick leave, Hospital days, Doctor visits
	• Nottingham Health Profile (NHP, part 1) scale: Physical mobility
	• Quality of Life Scale (QLS)
	• Life Fulfilment Scale
	
Psychological well-being	• Psychological General Well-being Scale (PGWB), except General Health scale
	• NHP scales: Emotional reactions, Social isolation, Energy.
	• Minnesota Multiphasic Personality Inventory-2 (MMPI-2) scale: Depression.
	• Hamilton Depression Scale (HDS).
	• Beck Depression Inventory (BDI).
	• Profile Of Mood States (POMS).
	• Sjöberg mood questionnaire.
	• State-Trait Anxiety Inventory (STAI) subscale: State anxiety.
	• Kellner Symptom Questionnaire (KSQ).
	• Symptom Checklist (SCL-90): Anxiety, Depression.
	• Mental Fatigue Questionnaire (MFQ).
	• Hospital Anxiety and Depression scale.
	
Health status	• General health questionnaire (GHQ).
	• NHP scales: Overall score, Pain, Sleep.
	• PGWB scale: General health.
	• Hopkins Symptom Checklist (HSCL).
	• Leisure time physical activity (VAS-score)
	• SCL-90 scale: Somatic complaints, Sleep.

## Results

Fifteen studies met our inclusion criteria for analysis of the effect of GH on patient-reported outcomes and were included in this meta-analysis. Study characteristics are shown in Table 2. Total number of patients is 830 and follow-up with a maximum of 24 months was analyzed.

**Table 2:** Included studies on GH therapy and psychological variables

**First author**, year [ref]	**N**	**Mean age (range) (years)**	**Duration therapy (months)**	**Trial design**	**Tests**
**Ahmad**, 2001 [28]	46	Unknown	3	Open	QoL-AGHDA
**Baum**, 1998 [29]	40	Median 51 (24–64)	18	Controlled	NHP, PGWB, GHQ, MMPI-2
**Burman**, 1995 [30]	36	46 (28–57)	9	Controlled	NHP, PGWB, HSCL
**Carroll**, 1997 [31]	38	42.9	6	Controlled	NHP, PGWB
**Cuneo**, 1998 [32]	83	41.2	12	Controlled-open	NHP
**Degerblad**, 1990 [33]	6	20–38	3	Controlled	Sjoberg, POMS
**Deijen**, 1998 [34]	48	27 (19–37)	24	Controlled-open	POMS vigor, STAI state, HSCL
**Giusti**, 1998 [35]	26	51 (21–74)	6	Controlled	KSQ, HDS
**Hernberg**, 2001 [36]	304	M: 50.8 F: 48.6	12	Open	QoL-AGHDA, VAS
**Murray**, 1999 [37]	65	38.7 (17–72)	8	Open	QoL-AGHDA, PGWB
**Sartorio**, 1995 [38]	8	29.6 (25–34)	6	Open	Dysphoria, Anxiety
**Soares**, 1999 [39]	9	39.4 (28–52)	12	Controlled-open	HDS, BDI
**Stouthart**, 2003 [9]	20	21 (17–27)	12	Open	QLS, STAI, POMS, SCL-90, HSCL
**Wallymahmed**, 1997 [40]	30	35	24	Controlled-open	NHP, MFQ
**Wiren**, 1998 [41]	71	45 (19–76)	24	Open	NHP, PGWB

BDI = Beck Depression Inventory; GHQ = General Health Questionnaire; HSCL = Hopkins Symptom Check list; HDS = Hamilton Depression Scale; MFQ = Mental Fatigue Questionnaire; MMPI-2 = Minnesota Multiphasic Personality Inventory-2; NHP = Nottingham Health Profile; KSQ = Kellner Symptom Questionnaire; PGWB = Psychological General Well Being Schedule; POMS = Profile of Mood States; SCL-90 = Symptoms Check List-90; Sjöberg = Sjöberg mood questionnaire; STAI = State-Trait Anxiety Inventory; QLS = Quality of Life Scale; QoL-AGHDA = Quality of Life Assessment of Growth Hormone Deficiency in Adults; VAS = Visual Analogue Scale.

**Figure 1:**
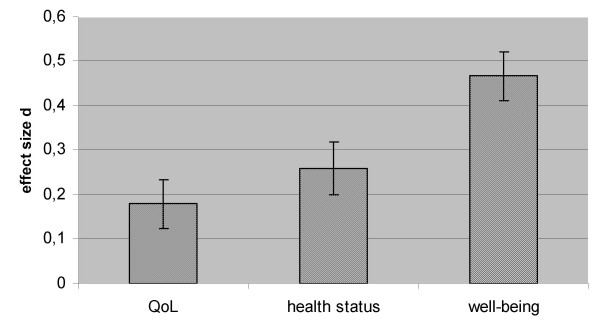
Average effect sizes for quality of life, health status and well-being.

A series of meta-analyses on open-label studies was carried out on our classification of instruments differentiating QoL, psychological well-being and health status. As the data were too limited to distinguish between different treatment lengths we analyzed the effects of pooled treatment durations. GH replacement with an average duration of 8.6 (±4.0) months (based on 26 *d*'s from 9 studies) improves QoL with a small effect size (*p *= 0.001; *d *= 0.18, 0.07–0.29 [CI]). With regard to psychological well-being after GH replacement with an average duration of 9.2 (±5.1) months (86 *d*'s from 13 studies) an increase with a medium effect size is found (*p *< 0.001; *d *= 0.47, 0.36–0.57 [CI]). Finally, GH replacement with an average duration of 9.4 (±4.0) months (31 *d*'s from 10 studies) increases health status with a small effect size (p < 0.001; *d *= 0.26, 0.14–0.37 [CI]). Thus, the largest effect is found for well-being, followed by health status and then Qol (Figure 1).

## Discussion

This present series of meta-analyses evaluated the effects of GH substitution on psychological parameters in GH deficient subjects, separately analyzed for QoL, psychological well-being and health status. Our aim was to examine whether GH would improve QoL, well-being and health status differently. Therefore we classified the psychological tests we encountered in the studies as being an instrument measuring QoL, well-being or health status.

After differentiating QoL from well-being and health status we determined the effect sizes obtained from the meta-analyses on GH replacement. GH replacement appeared to improve well-being the most, followed by health status and then QoL. The effect sizes indicate that the effect on well-being is more than twice as large as that concerning QoL and nearly twice as large as that concerning health status. It may be argued that this result may be associated with differences in the psychometric properties of the scales for QoL, well-being and health status. However, an effect size is a standardized, dimensionless number, which allows the comparison of the results of different studies or the results of different tests within one study [26]. In short, effect size is a simple quantitative measure that provides one useful index of the importance of an effect. In addition, a distinction can be made between 'small', 'medium' and 'large' effect sizes. A medium effect size is conceived as one large enough to be visible to the naked eye [21]. Thus, particularly the medium effect size of well-being is substantial while the effect sizes of health status and QoL seem to be of less importance. This suggests that the generally reported effects of GH replacement on QoL may be overestimated and actually reflect the effect on psychological well-being. However, as the effect size index only pertains to statistical effects our data are not indicative of the clinical relevance of the present results. In order to determine clinical relevance we should have been looking at the minimum clinically important difference for each scale. Unfortunately, such a clinical determination was not possible because of the variety of instruments. We can only conclude that a larger effect size may be associated with a more important clinical effect, but this needs not necessarily be the case. In contrast, it is even conceivable that the larger effect size observed for well-being reflects a smaller clinical relevance than the small effect size observed for QoL.

Finally, it is important to note that a positive effect of GH on psychological outcomes has been found mainly in open studies lacking a control group. The effects of GH treatment on patient reported outcomes have been compared to placebo in a meta-analysis we performed earlier on the same database [27]. The purpose of that analysis was to determine whether GH replacement has any beneficial effect on psychological variables. Therefore, the data of all questionnaires were pooled disregarding measuring QoL, well-being or health status. GH treatment effects on these averaged psychological functions were then compared with placebo. The overall effect of GH treatment with a median duration of 6 months was found not to be better than placebo. After reporting these data we decided to perform the present meta-analysis with a quite different objective, that is identifying which variable may be most sensitive to the effects of GH treatment.

It cannot be excluded that the enhanced well-being we observed in open studies in addition to the absence of a difference between GH treatment and placebo point to placebo-effects. The observed improvement in psychological outcomes in the open studies can therefore be attributed to other factors than GH. The attention and care given to the patients with GHD in the open studies may improve patient-reported outcomes more substantially than the contribution of GH itself.

At this point it is important to note that the present meta-analysis is based only on a selected subgroup of studies, because a lot of studies did not meet our inclusion criteria. For instance, in spite of pooling, our sample size appeared still to be quite small (i.e. 830 patients). In addition, the variability of patient characteristics, the diversity in study designs including the variety of dependent variables and publication bias may distort the results. A number of moderator or confounding variables may have attributed to the variance in effect sizes. These variables can be assumed to be sex, age, medial history (radiotherapy), dose of GH and severity or type of GHD. The limited amount of data pertaining to these confounders including the differential treatment effects in patients with either childhood-onset/adulthood-onset GHD or isolated GHD/multiple pituitary hormone deficiencies did not allow to control for these confounders.

The present meta-analysis may therefore have lead to unjustified conclusions. However, with respect to publication bias, it is known that especially reports lacking positive treatment effects are not published. Thus, if such studies had been published and be part of the meta-analysis, they would have resulted in smaller effect sizes than those reported here.

## Conclusion

From the present meta-analysis we may conclude that the psychological effects of GH treatment are not quite clear yet. A variety of instruments have been used to determine specific effects on QoL, well-being and health status. However, up until now the instruments have not been reliably classified into those measuring QoL, well-being or health status. The inconsistent classification of psychological questionnaires may have lead to unjustified conclusions concerning the psychological effects of GH therapy. The results of the present meta-analysis based on generally accepted definitions of these concepts indicate that the effects of GH treatment are most obvious with respect to well-being, followed by health status and QoL. It may thus well be true that the frequently reported effects of GH on QoL are overvalued. Therefore, to get more insight into the precise nature of the psychological effects of GH therapy we recommend that in future studies more uniform classifications of psychological outcomes should be used.

## Authors' contributions

JBD and LIA made substantial contributions to conception and design, acquisition, analysis, and interpretation of data and writing the manuscript. JW was involved in the design of the study and data acquisition, performed the statistical analysis and participated in drafting the manuscript. MLD was involved in interpretation of data and in revising the manuscript. All authors read and approved the final manuscript.
